# Laparoscopic vs. Abdominal Radical Hysterectomy for Locally Advanced Cervical Cancer

**DOI:** 10.3389/fonc.2019.01331

**Published:** 2019-11-27

**Authors:** Wenhui Wang, Lei Li, Ming Wu, Shuiqing Ma, Xianjie Tan, Sen Zhong

**Affiliations:** Department of Obstetrics and Gynecology, Peking Union Medical College Hospital, Beijing, China

**Keywords:** locally advanced cervical cancer, radical hysterectomy, survival, neoadjuvant chemotherapy, mini-invasive surgery, open surgery

## Abstract

This study is to compare the survival outcomes of laparoscopic radical hysterectomy (LRH) to those of abdominal radical hysterectomy (ARH) for patients with locally advanced cervical cancer (LACC). Patients with the International Federation of Gynecology and Obstetrics (FIGO) 2009 stage IB2 to IIB LACC who underwent radical hysterectomy between 2001 and 2015 were identified. The disease-free survival (DFS) and overall survival (OS) were compared according to the surgical approach and were adjusted based on clinicopathologic characteristics. A total of 396 patients were included in the study, with 179 (45.2%) and 217 (54.8%) patients in the ARH and LRH groups, respectively. The LRH group showed a significantly lower amount of estimated blood loss, lower blood transfusion rate and shorter length of hospital stay. Overall, there were no significant differences in the 5-year DFS and 5-year OS between the LRH and ARH groups with the Kaplan-Meier method. However, multivariate analyses identified LRH as an independent prognostic factor for a poor DFS (hazard ratio [HR] 2.5; 95% confidence interval [95% CI] 0.19 to 0.87; *p* = 0.02). The analysis of stage IB2 disease and the squamous subtype (61.9% and 87.9% of all participants, respectively) reached the same conclusion. When stratifying by FIGO stage, the patients with IB2 (*n* = 348) in the ARH group had a significantly better DFS (HR 0.14, 95% CI 0.05–0.42, *p* < 0.01) and OS (HR 0.17, 95% CI 0.04–0.67, *p* = 0.11) than those in the LRH group in the Cox regression model. However, no differences were found in patient with stage IIA1, IIA2, or IIB in Cox regression model. When stratifying by histological types, for the patients with squamous carcinomas (*n* = 375), in Cox model, ARH had a significantly superior DFS compared with those who underwent LRH (HR 0.45, 95% CI 0.25–0.82, *p* = 0.01), but the OS was not statistically significant (HR 0.57, 95% CI 0.27–1.20, *p* = 0.14). However, no differences were found in patient with adenocarcinoma and adenosquamous carcinomas in the Cox model. Therefore, ARH was associated with a higher DFS than LRH in patients with LACC, especially in patients with stage IB2 disease or the squamous subtype.

## Introduction

Cervical cancer (CC) is one of most common cancer-related deaths among women worldwide (1). China also has a high burden of disease and a relatively high prevalence of advanced-stage disease (2). Locally advanced cervical cancer (LACC) refers to stage IB2, IIA, and IIB carcinomas, classified by the International Federation of Gynecology and Obstetrics (FIGO) staging system (3). The treatment for women with LACC remains challenging. The standard treatment for LACC is cisplatin-based concurrent chemoradiotherapy (CCRT) (4, 5). However, surgical treatment, including radical hysterectomy (RH) followed by adjuvant treatment is also a preferred treatment option for LACC in some circumstances (6, 7) and could offer potential benefits, including reducing the burden of tumor, preserving ovarian function and precisely determining the postoperative stage on the basis of histopathologic findings, thereby allowing quality of life improvements in young patients and individualizing postoperative treatment (4).

Recently, a phase III trial (8) and an epidemiological study (9) revealed that women undergoing minimally invasive surgery (MIS) through RH for early CC had a higher recurrence and worse survival than those who underwent abdominal radical hysterectomy (ARH). Nevertheless, only a few studies have compared the surgical and oncologic outcomes of MIS through RH to those of ARH for LACC. In the report from Zanagnolo et al. (10), ARH and robotic RH after neoadjuvant chemotherapy (NAC) in women with LACC were associated with similar perioperative and oncologic outcomes. Type B RH after NAC in well-selected patients is a safe procedure that could potentially reduce the operative time and late postoperative morbidity, without detrimental effects to survival (11). However, these trials lack sufficient sample sizes, long-term follow-ups and good comparators, which limit the generalizability of their conclusions.

In this study, we aimed to compare the survival outcomes of LRH with those of ARH for patients with FIGO stage IB2 to IIB CC at a tertiary institutional hospital in China from 2001 to 2015. All major procedures, consisting of parametrium resection and systematic lymphadenectomy, were performed by the corresponding authors.

## Methods

### Ethical Approval

The Institutional Review Board from the study center approved the study (No. ZS-1427). All patients provided consent before treatment. The registration number is NCT03291236 (*clinicaltrials.gov*). All procedures performed in the study involving human participants were in accordance with the ethical standards of the institutional and/or national research committee and with the 1964 *Declaration of Helsinki* and its later amendments or comparable ethical standards.

### Study Design and Patient Enrollment

A total of 428 patients with CC classified as stage IB2 to IIB, according to the FIGO staging system of 2009, who received class III or type C ARH or LRH based on the Q-M classification from February 2, 2001 to November 11, 2015 at the study center were enrolled in this retrospective study. The inclusion criteria consisted of the following: FIGO stage IB2 to IIB cancer diagnosed by pelvic examinations conducted by two experienced physicians of gynecologic oncology; histopathologically proven primary cervical squamous carcinoma, adenocarcinoma or adenosquamous carcinoma; aged 18 years or older; and Eastern Cooperative Oncology Group performance status scores of 0 or 1. Patients were excluded if they had distant metastasis based on presurgical imaging.

The patients were divided into ARH and LRH groups according to their definitive surgical route. The primary objective was to compare the disease-free survival (DFS) and overall survival (OS) between the two groups. The secondary endpoints included the surgical outcomes of the two groups.

### Treatments and Follow-up

Surgical treatment consisted of LRH and ARH, with bilateral salpingo-oophorectomy, and lymphadenectomy of the pelvic lymph nodes (PLNs) and para-aortic lymph nodes (PALNs). Salpingectomy was performed in young patients, along with translocation of the ovaries to the peritoneum above the level of the anterior superior spine. All the major surgical procedures (resection of parametrium and systematic lymphadenectomy) were primarily performed by the corresponding authors, and the choice of LRH and ARH was due to the experiences and learning curve of the surgeons. The surgical extent followed the specifications of class III of the Piver classification (before 2011) (12) or type C of the Q-M classification (after 2011) (13, 14). The nerve-sparing RH procedures have been described in another study (15). The complications related to ARH and LRH that occurred within 3 months were reviewed and collected from the medical records as adverse events according to the Common Terminology Criteria for Adverse Events (CTCAE) v4.03 (16). As currently there were no criteria or standard for utilizing NAC, NAC was given for fractional patients with bulky tumors after a discussion with the patients. The NAC protocols consisted of TC (paclitaxel 175 mg/m^2^, carboplatin AUC 5 on day 1 in a 21-day cycle administered via intravenous infusion), TP (paclitaxel 175 mg/m^2^, cisplatin 70 mg/m^2^ on day 1 of a 21-day cycle via intravenous infusion) or PF (fluorouracil 1,000 mg/m^2^ on days 1 to 4, cisplatin 70 mg/m^2^ on day 1 of a 28-day cycle via intravenous or transuterine arterial infusion). Postoperative adjuvant therapies were provided for the eligible patients and included systematic chemotherapy, radiotherapy, radiotherapy and concurrent chemoradiotherapy (CCRT) or a combination of these regimens. The administration of all adjuvant therapies followed the relevant contemporary guidelines (17).

All tumor specimens were investigated with detailed pathological examinations used to determine the characteristics of the pathological subtypes, lymphovascular space invasion (LVSI), invasion depth of the stroma, lymphatic metastasis, involvement of the uterus or parametrium, and status of the incision margin. For RH performed before 2009, the staging was reviewed and redefined according to FIGO 2009 criteria (18).

All patients were followed until March 1, 2017. The patients were closely followed up according to the customized protocol: all patients were asked to visit an outpatient clinic every 3 months for the first year, every 4 months for the second year, every 6 months for the third year, and every year for the remainder of the follow-up time. The patients underwent physical examinations, cytology tests, and imaging evaluations. Recurrence was validated by imaging examination and/or biopsy. The recurrent sites were divided into categories including within the pelvic cavity and at distant sites. Mortality was confirmed by reviewing the medical records and interviews by telephone and/or email. DFS was defined as the length of time after receiving primary treatment for a cancer that a patient lived without any signs or symptoms of that cancer, and OS was defined as the length of time that the patient was alive after receiving primary treatment for that cancer. Specifically, the survival analysis was performed again in patients of stage IB2 to IIA2.

### Statistical Analysis

SPSS 22.0 (SPSS Inc., Chicago, IL, USA) was used for statistical analysis. The characteristics of different patients were compared between the ARH and LRH groups using Student's *t*-tests, Wilcoxon rank sum tests and chi-square tests. The Kaplan-Meier method with log-rank tests was used to compare the survival outcomes between the two groups as univariate methods. In multivariate analyses, the hazard ratios (HRs) and 95% confidence intervals (CIs) were calculated using Cox proportional hazards regression models. The following factors were used to adjust the hazard ratios of survivals between ARH and LRH groups in a Cox regression model: operative period (before and after 2010), menstrual status, nerve-sparing radical hysterectomy (NSRH), FIGO stage, pathological subtype, differentiation status, lymph node metastasis, involvement of the parametrium and vaginal margin, invasion depth of stroma, LVSI, residual lesions, postoperative complications and adjuvant therapies. All reported statistical significances were two-tailed at a level of 0.05.

## Results

### Baseline Clinical and Pathological Characteristics

**Figure 1:**
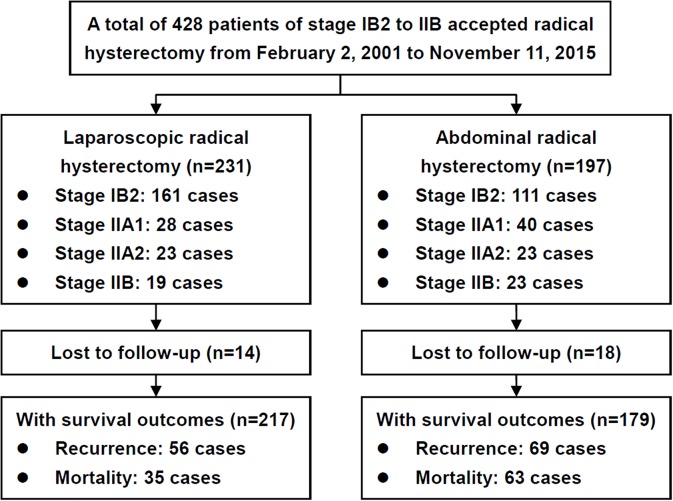
Flow diagram of the study.

During the study period, 428 patients were included, and 396 patients had definitive survival outcomes (Figure 1; Supplement File 1). The baseline clinical and pathological characteristics are presented in Table 1. In total, 179 patients (45.2%) underwent ARH, and 217 patients (54.8%) underwent LRH; the patients had mean ages of 45.95 years (*SD* = 7.33) and 44.76 years (*SD* = 7.74), respectively. No conversion from LRH to ARH occurred. Most of the baseline and pathological characteristics between these two groups were well-balanced, including preoperative NAC and postoperative adjuvant therapy. However, the patients in the ARH group presented with a more advanced FIGO stage (*p* = 0.027), earlier surgical period (*p* < 0.001), smaller tumor size (*p* = 0.011) and poorer differentiation (*p* < 0.001) than those in the LRH group.

**Table 1:** Clinical and pathological characteristics of patients in the LRH and ARH groups.

	**ARH (179)**	**LRH (217)**	***p***
Age (year) (mean ± SD)	45.95 ± 7.331	44.76 ± 7.743	0.121
BMI (Kg/m^2^) (mean ± SD)	23.56 ± 3.286	23.25 ± 2.629	0.289
Menopause n (%)	57 (31.8)	49 (22.6)	0.038
ECOG status			0.312
0	155 (86.6)	195 (13.4)	
1	24 (13.4)	22 (10.1)	
Max diameter of tumor (mm) (mean ± SD)	43.32 ± 11.886	46.37 ± 11.543	0.011
FIGO stage *n* (%)			
IB2	97 (54.2)	148 (68.2)	0.027
IIA1	40 (22.3)	28 (12.9)	
IIA2	23 (12.8)	23 (10.6)	
IIB	19 (10.6)	18 (8.3)	
Duration of surgery (min) (mean ± SD)	223.02 ± 41.021	206.36 ± 41.598	<0.001
Estimated blood loss (ml) (mean ± SD)	476.82 ± 302.390	304.61 ± 323.110	<0.001
Blood transfusion *n* (%)	36 (20.1)	14 (6.5)	<0.001
Hospital stay (days) (mean ± SD)	19.42 ± 13.223	14.11 ± 7.598	<0.001
Grade 3/4 complications^*^n (%)	22 (12.3)	6 (2.8)	<0.001
Without residual lesions *n* (%)	113 (63.1)	117 (53.9)	0.386
No. of lymph nodes resected (mean ± SD)	36.65 ± 11.66	41.62 ± 12.92	<0.001
Pathologic subtype *n* (%)			0.012
Squamous carcinoma	160 (89.4)	188 (86.6)	
Adenocarcinoma	9 (5.0)	25 (11.5)	
Adenosquamous carcinoma	10 (5.6)	4 (1.8)	
Differentiation of tumor *n* (%)			<0.001
G1	7 (3.9)	28 (12.9)	
G2	77 (43.0)	109 (50.2)	
G3	95 (53.1)	80 (36.9)	
Invasion depth of stroma *n* (%)			0.280
<1/3	70 (39.1)	87 (40.1)	
>1/3 but <2/3	64 (35.8)	63 (29.0)	
>2/3	45 (25.1)	67 (30.9)	
Positive LVSI *n* (%)	71 (39.7)	87 (40.1)	0.931
Parametrial involvement *n* (%)	19 (10.6)	29 (13.4)	0.404
Positive vaginal margin *n* (%)	20 (11.2)	14 (6.5)	0.095
Metastasis to lymph nodes *n* (%)	31 (17.3)	43 (19.8)	0.526
NSRH *n* (%)	17 (9.5)	92 (42.4)	<0.001
Postoperative adjuvant therapy *n* (%)	166 (92.7)	207 (95.4)	0.261
NAC *n* (%)	123(68.7)	132(60.8)	0.103
Response to NAC (%)	84/123 (68.3%)	85/132 (64.4%)	0.511
Operative period *n* (%)			<0.001
2010 and before	146 (81.6)	40 (18.4)	
After 2010	33 (18.4)	177 (81.6)	

* *These complications and their severity were defined as adverse events happened within 3 months after RH according to Common Terminology Criteria for Adverse Events (CTCAE) v4.03*.

*BMI, body mass index; FIGO, International Federation of Gynecology and Obstetrics; G1, G2 and G3, grade of the differentiation; LRH, laparoscopic radical hysterectomy; LVSI, lymph-vascular space invasion; ARH, abdominal radical hysterectomy; NSRH, nerve-sparing radical hysterectomy; SD, standard deviation; NAC, Neo-adjuvant chemotherapy*.

Specifically, 123 (68.7%) and 132 (60.8%) patients in the ARH and LRH groups received NAC (*p* = 0.103), and 84 (68.3%) and 85 (64.4%) patients achieved partial or complete response (*p* = 0.511), respectively. There were no significant differences in the NAC protocols and administration route between the two groups (all *p*-values > 0.05).

### Survival Outcomes

The median follow-up was 41.3 months (range 6–193.5), the 5 and 10-years DFS of the whole population were 70 and 60%, and the 5 and 10-years OS rates were 75% and 68%, respectively. In the ARH and LRH groups, there were 69 (38.5%) and 56 recurrences (25.8%, *p* = 0.007), and 63 (35.2%) and 35 deaths (16.1%, *p* < 0.001), respectively. Among the recurrent cases, 46 of 69 (66.7%) cases in the ARH group and 35 of 56 (62.5%) cases in the LRH group occurred within the pelvic cavity (*p* = 0.628). However, vaginal vault recurrence occurred in 14 of 69 (20.3%) patients of the ARH group and 4 of 56 (7.1%) of the LRH group (*p* = 0.037), respectively. In Kaplan-Meier analysis, the DFS and OS were related with several clinicopathological risk factors, including operative period (before and after 2010), menstrual status, nerve-sparing radical hysterectomy (NSRH), FIGO stage, pathological subtype, differentiation status, lymph node metastasis, involvement of the parametrium and vaginal margin, invasion depth of stroma, LVSI, residual lesions, postoperative complications and adjuvant therapies (all *p* <0.05).

**Figure 2:**
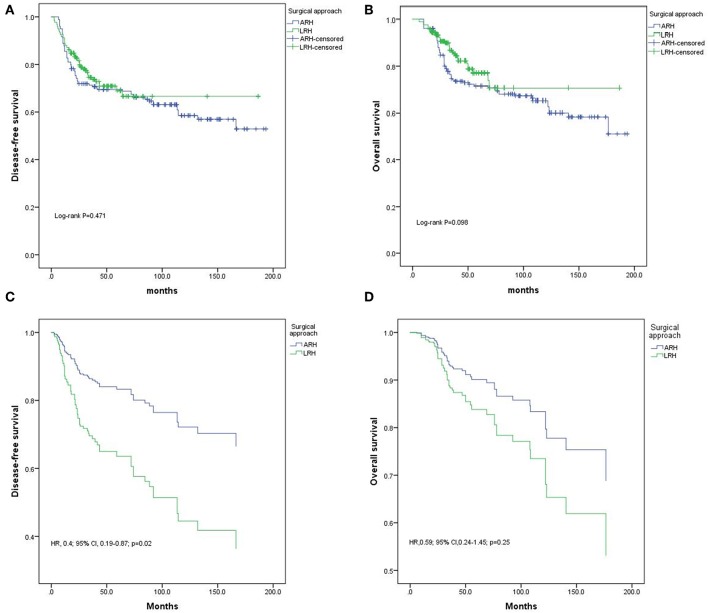
Disease-free survival (DFS) and overall survival (OS) of the whole population. **(A)** DFS results by the Kaplan-Meier method; **(B)** OS results by the Kaplan-Meier method; **(C)** DFS results by Cox regression analysis; **(D)** OS results by Cox regression analysis.

The 5-year OS rates in the ARH and LRH groups were 71% and 77% (*p* = 0.10, Figure 2A), and the 5-year DFS rates were 69 and 69% (*p* = 0.47 Figure 2B), respectively. However, multivariate analyses of the Cox regression model identified ARH as an independent protective prognostic factor for DFS (HR 0.4; 95% CI 0.19–0.87; *p* = 0.02, Figure 2C). There were no significant differences in OS (HR 0.59; 95% CI 0.24–1.45; *p* = 0.25, Figure 2D) between the two groups.

Specifically, in our study, NAC did not provide a more superior DFS or OS in the whole population, in the LRH and ARH groups, or in patients with various stages of disease in the multivariate analyses compared with non-NAC patients in Cox regression model (Supplement Table 1).

In particular, ARH still had more superior DFS but not OS than LRH in 386 patients of stage IB2 to IIA2 (i.e., excluding stage IIB patients) in Cox regression model: DFS, HR 0.2, 95% CI 0.1–0.5, *p* < 0.001; OS, HR 0.5, 95% CI 0.2–1.3, *p* = 0.169.

#### Survival Analysis of Different FIGO Stages

**Figure 3:**
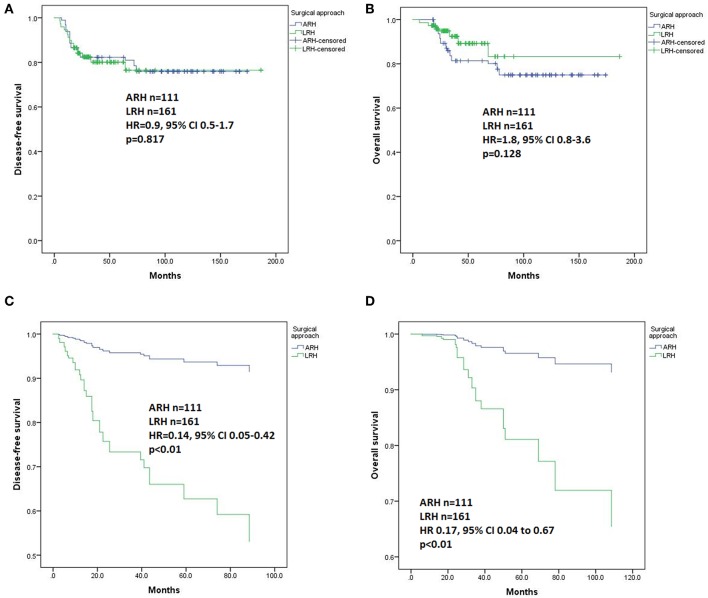
Disease-free survival (DFS) and overall survival (OS) of the patients with stage IB2. **(A)** DFS results by the Kaplan-Meier method; **(B)** OS results by the Kaplan-Meier method; **(C)** DFS results by Cox regression analysis; **(D)** OS results by Cox regression analysis.

For patients with stage IB2 (*n* = 272), Kaplan-Meier analysis revealed no differences in DFS (*p* = 0.817, Figure 3A) or OS (*p* = 0.128, Figure 3B). In the Cox regression model, the patients in the ARH group had a significantly better DFS (HR 0.14, 95% CI 0.05–0.42, *p* < 0.01, Figure 3C) and OS (HR 0.17, 95% CI 0.04–0.67, *p* < 0.01, Figure 3D) than those in the LRH group.

**Figure 4:**
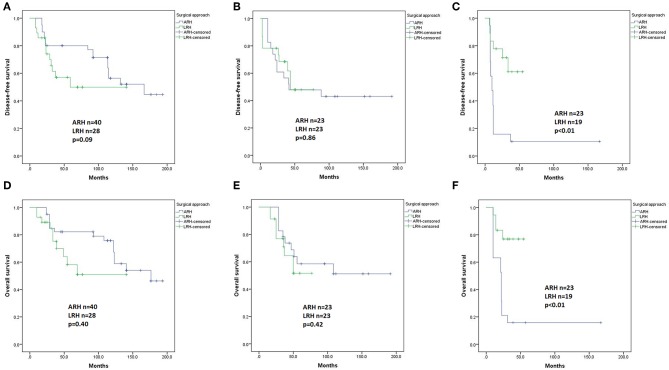
Disease-free survival (DFS) and overall survival (OS) of the patients with stage IIA1, IIA2, and IIB disease by the Kaplan-Meier method. **(A)** DFS of stage IIA1 patients; **(B)** DFS of stage IIA2 patients; **(C)** DFS of stage IIB patients; **(D)** OS of stage IIA1 patients; **(E)** OS of stage IIA2 patients; **(F)** OS of stage IIB patients.

For patients with IIA1 (*n* = 68) and IIA2 (*n* = 46) disease, Kaplan-Meier analysis revealed no differences in DFS (*p* = 0.09 and 0.86, respectively, Figures 4A,B) or OS (*p* = 0.40 and 0.42, respectively, Figures 4D,E) between the LRH and ARH groups. However, for patients with stage IIB disease (*n* = 42), the ARH group had a significantly worse DFS and OS than the LRH group (*p* < 0.01, Figures 4C,F). However, they all had no significant differences in the Cox regression model.

#### Survival Analysis of Different Histological Subtypes

**Figure 5:**
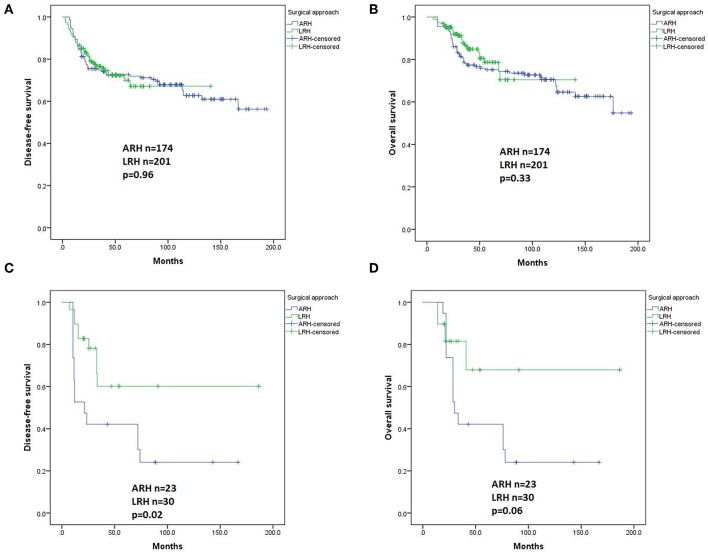
Disease-free survival (DFS) and overall survival (OS) of the patients with different histological subtypes by the Kaplan-Meier method. DFS **(A)** and OS **(B)** of patients with squamous carcinomas; DFS **(C)** and OS **(D)** of patients with adenocarcinomas plus adenosquamous carcinomas.

In univariate analysis, in patients with squamous carcinomas (*n* = 375), there were no significant differences in DFS (*p* = 0.96, Figure 5A) or OS (*p* = 0.33, Figure 5B) based on the type of surgery. In Cox regression model, in patients with squamous carcinomas, those who underwent ARH had a significantly superior DFS compared with those who underwent LRH (HR 0.45, 95% CI 0.25–0.82, *p* = 0.01), but the OS was not statistically significant (HR 0.57, 95% CI 0.27–1.20, *p* = 0.14).

In patients with adenocarcinoma and adenosquamous carcinomas (*n* = 53), the ARH group showed a worse DFS and worse OS than the LRH group (*p* = 0.02 and *p* = 0.06, Figures 5C,D respectively). However, the difference disappeared in the Cox regression model.

### Surgical Outcomes

Compared with the ARH group, the LRH group had better surgical outcomes, including a significantly lower amount of estimated blood loss, lower blood transfusion rate, shorter operation time and hospital stay, more LNs resected, higher NSRH rate and lower grade 3/4 complications (*p* < 0.001).

## Discussion

In this study, we reported a large cohort of LACC patients who underwent RH with or without NAC. The surgical and survival outcomes between ARH and LRH could become the basis of decision making for physicians and patients, which could probably improve the quality of life of these relatively young patients. In the United States, CCRT is typically preferred over radical surgery for patients with LACC (17). However, to reduce the size of the primary tumor (19), improve the chances of operative curability and safety, and reduce long-term morbidity due to radiotherapy (20), NAC followed by RH had been attempted for patients with LACC. As previously reported, NAC followed by RH was suggested as a useful strategy (21), even for patients with nonsquamous cell carcinoma of the uterine cervix (22). Currently, no conclusive evidence is available regarding the relative benefits and drawbacks of primary RH vs. primary chemoradiotherapy for LACC (23). In a phase 3 randomized controlled study of the squamous subtype of LACC, NAC followed by chemoradiotherapy could improve DFS but not the OS compared with NAC followed by RH, and NAC had more toxicity (24). Judged by these findings, the impact of RH on the surgical or oncologic benefits in patients with LACC still requires more substantial evidence.

In our study, compared to LRH, ARH had a significantly superior survival outcome, especially in terms of DFS. The non-significant differences in OS between the two groups may be due to the insufficient follow-up periods in the LRH group. These findings are in accordance with previously reported results of early stage cervical cancer (8, 9). In a subgroup analysis, ARH for stage IB2 and the squamous carcinoma subtype led to a better DFS than LRH did, mainly because the ARH patients comprised the majority of the study population. However, despite several assumptions, the reasons for the inferior survival outcomes of MIS are unknown.

The learning curves of RH were most likely the most important reason, since almost all the LRH procedures were performed after 2011, and all the ARH procedures were performed before 2011. Due to the complex and complicated characteristics of radical surgeries, a lengthy learning process is needed to achieve comparable survival outcomes, which is equally essential for laparotomy or laparoscopy (25). Surgeons in the United States began to adopt minimally invasive RH for the treatment of cervical cancer in 2006 (26). In recent published study, the adoption of MIS was associated with a significant change in the survival trends and coincided with the beginning of a decline of survival rate between 2006 and 2010 (9). No definite reasons could explain this phenomenon except for the surgical route, and very few studies have considered factors that involve surgeons and their learning curves (25). Mastery of LRH requires experience with at least 20 cases and up to 50 cases (27–29), which shows the gradual slope of the learning curve (29). The learning curve for LRH and LN dissection would reach a turning point at 40 cases (30). These studies support the essential evaluation of the learning curves of a demanding surgical modality, both in early stage cervical cancer and LACC.

Other perspectives in our study need further consideration. A better description of the surgical procedure for the purpose of quality control is essential for future studies, since no conversions occurred in our study, which differs from other reports with rates of 1.5–3.5% (31). The significantly higher recurrence at the vaginal vault in the ARH group was comparable to the previously reported results (8). It is supposed that total laparoscopic/robotic intracorporeal colpotomy under CO_2_ pneumoperitoneum might pose a risk for implantation in vaginal cuff margin and intraperitoneal spread (32). ARH may limit the chance of intra-abdominal seeding during cancer cell removal by placing two sets of clamps across the vagina below the cervix (33). However, all of these considerations lack the support of validated evidence and cannot explain the poor survival outcomes of LRH.

Studies suggest that histopathology is an independent prognostic factor that plays an essential role in the outcomes of LACC patients (34). Most reports suggest that patients with adenocarcinoma and adenosquamous carcinoma have a worse prognosis than patients with squamous carcinoma (34). In our study, there was a meaningful survival difference between the ARH and LRH groups in patients with squamous carcinoma, which is the primary subtype. The results indicate that further exploration of other less common histological subtypes is needed. At present, based on our findings, ARH should be the procedure of choice for LACC, at least for the squamous subtype.

In our study, as expected, LRH resulted in better surgical outcomes than ARH, including a shorter mean operating time, less blood loss, and shorter hospital stay, all of which are in accordance with previously reported findings (23). However, these surgical benefits still could not compensate for the low survival. A long, comprehensive evaluation of the surgical protocols is essential to benefit patients.

In our study, NAC did not provide survival benefits in the whole cohort or in the subgroups of various surgical routes or stages. Despite of numerous cohort studies guaranteeing the safety and effectiveness of NAC, randomized controlled trials (35) and meta-analyses (36) have proved that NAC had no positive impact on the survival outcomes of patients with LACC. Therefore, the applicability of NAC in LACC is controversial (17). However, current randomized studies of NAC did not rigorously follow uniform chemotherapy protocols (35). Currently, some expert opinions provided the appropriate selection of patients for the application of NAC (37).

The strengths of our study were the relatively large cohort and uniform RH procedures performed by experienced physicians. However, there are several major limitations. Similar to previous studies about LACC, our conclusion was also limited by inadequate power and probable residual confounding. As patients were all from a single center and a single team, selection bias would probably greatly interfere with the interpretation of the oncologic outcomes. The lack of uniform NAC protocols also limits the conclusions on the influence of NAC. Data on long-term survival assessed in randomized trials or in large cohort studies are needed for patients with LACC. The insufficient number of disease cases from stages other than IB2 and subtypes other than squamous carcinoma also limits the generalizability of our conclusions. Last, the inclusion of patients with stage IIB did not accord with current guideline (17). We have performed separate analysis for stage IB2 to IIA2, and have achieved similar conclusions. However, we wish our data, failure or possible experiences would provide knowledge and insight on the topic of treatment for LACC, the troublesome and confusing type.

## Conclusions

Despite its favorable surgical outcomes, LRH was associated with a shorter DFS than ARH for patients with LACC (IB2 to IIB) as a whole population, and in patients with stage IB2 disease or the squamous subtype. However, in patients with stage IIA and IIB, and in patients of non-squamous subtypes, LRH hand no significant differences with ARH in the survival prognosis, probably due to the limited sample size in these subgroups. NAC had no significant impact on the survival outcomes.

## Data Availability Statement

All datasets generated for this study are included in the article/Supplementary Material.

## Ethics Statement

The Institutional Review Board of Peking Union Medical College Hospital approved this study (No. ZS-1427). The registration No. is NCT03291236 (*clinicaltrials.gov*).

## Author Contributions

LL and MW conceived of the original idea for the study, interpreted the results, carried out the statistical analysis, edited the paper, and were the overall guarantors. MW completed all the major procedures of the radical hysterectomy, WW obtained ethical approval, contributed to the preparation of the data set, interpreted the results and contributed to drafts of the paper. SZ, SM, and XT contributed to the study design, interpretation of the results, and commented on drafts of the paper.

### Conflict of Interest

The authors declare that the research was conducted in the absence of any commercial or financial relationships that could be construed as a potential conflict of interest.

## Supplementary Material

Supplement Table 1 Survival outcomes of patients with and without NAC followed by RH and systematic lymphadenectomy and the stratified analysis according to stages in Cox regression model.

Supplement File 1 Raw data of the study.

## Abbreviations

95% CI: 95% confidence interval
ARH: abdominal radical hysterectomy
CC: cervical cancer
DFS: disease-free survival
HR: hazard ratio
LACC: locally advanced cervical cancer
LRH: laparoscopic radical hysterectomy
MIS: minimally invasive surgery
NAC: neoadjuvant chemotherapy
OS: overall survival.
