# Supplementary material for: Laparoscopic vs. Abdominal Radical Hysterectomy for Locally Advanced Cervical Cancer
Source: Front Oncol. 2019 Nov 27;9:1331. doi: 10.3389/fonc.2019.01331 (PMC6890871; doi:10.3389/fonc.2019.01331)
Supplement: Supplement Table 1 — Survival outcomes of patients with and without NAC followed by RH and systematic lymphadenectomy and the stratified analysis according to stages in Cox regression model. [file Table_1.docx]

Supplement Table 1

Survival outcomes of patients with and without NAC followed by RH and systematic lymphadenectomy and the stratified analysis according to stages in Cox regression model.

| **n (%)** | **Primary RH group (n=125)** | **NAC+RH group (n=271)** | **P value** |
| --- | --- | --- | --- |
| Recurrence | 35 (24.6%) | 91 (35.7%) | 0.025 |
| HR of DFS (95% CI) |  |  |  |
| Total | Reference | 1.4 (0.9-2.0) | 0.134 |
| IB2 (n=245) | Reference | 1.6 (0.9-3.1) | 0.116 |
| IIA1 (n=68) | Reference | 1.0 (0.4-2.1) | 0.915 |
| IIA2 (n=46) | Reference | 1.7 (0.6-4.7) | 0.273 |
| IIB (n=37) | Reference | 0.5 (0.2-1.3) | 0.158 |
| Mortality | 29 (20.4%) | 69 (27.1%) | 0.147 |
| HR of OS (95% CI) |  |  |  |
| Total | Reference | 1.0 (0.7-1.6) | 0.876 |
| IB2 (n=245) | Reference | 0.9 (0.4-1.8) | 0.717 |
| IIA1 (n=68) | Reference | 1.0 (0.4-2.4) | 0.984 |
| IIA2 (n=46) | Reference | 1.8 (0.6-5.5) | 0.282 |
| IIB (n=37) | Reference | 0.4 (0.2-1.1) | 0.073 |

NAC, neo-adjuvant chemotherapy. OS, overall survival. DFS, disease free survival. RH, radical hysterectomy.
